# What Comes First Metacognition or Negative Emotion? A Test of Temporal Precedence

**DOI:** 10.3389/fpsyg.2019.02507

**Published:** 2019-11-19

**Authors:** Lora Capobianco, Calvin Heal, Measha Bright, Adrian Wells

**Affiliations:** ^1^Faculty of Biology, Medicine and Health, School of Health Sciences, Division of Psychology and Mental Health, The University of Manchester, Manchester, United Kingdom; ^2^Greater Manchester Mental Health NHS Foundation Trust, Manchester, United Kingdom; ^3^Faculty of Biology, Medicine and Health, School of Health Sciences, Division of Population Health, The University of Manchester, Manchester, United Kingdom

**Keywords:** metacognitive beliefs, distress, cross-lagged analysis, structural equation modeling, S-REF

## Abstract

The Self-Regulatory Executive Function model predicts that emotional symptoms and metacognition can causally affect each other. Crucially, for the model metacognition must cause emotion disorder symptoms. Therefore, in time-series data involving repeated measurements, metacognitions should predict subsequent changes in emotion. 265 participants completed a questionnaire battery three times over a 2 month period. Structural equation modeling (SEM) using cross-lagged panel analysis tested the inter-relationships between metacognitive beliefs, anxiety and depression symptoms over time. The cross-lagged structural model was a significantly better fit than the autoregressive model. Metacognitive beliefs were found to predict subsequent symptoms of anxiety while symptoms of anxiety predicted later metacognition over different time courses. The metacognition factor representing uncontrollability and danger of thoughts appeared to be prominent in the effects observed. Metacognitions and depression were also positively related over time to a lesser degree, but in the cross-lagged model these temporal relationships were non-significant. This is likely due to low levels of depression within the sample and low variability over time. The findings for anxiety are consistent with the S-REF model and with experimental and prospective studies supporting metacognitive beliefs as a causal mechanism in psychological distress symptoms.

## Introduction

A crucial question in formulating the role of metacognitive factors in emotional symptoms concerns whether or not these factors have a causal or contributory role or merely represent an effect of such dysfunction. The Self-regulatory executive function model (S-REF; Wells and Matthews, 1994, 1996) proposes that specific metacognitions increase emotional dysfunction by, for example, interacting with environmental factors and giving rise to a pattern of extended negative thinking in response to stress. Thus, metacognition should precede symptoms in causal time-series data. Never the less, the model also allows for reciprocal causation, in which emotion can also impact on metacognition. For example, some anxiety or mood symptoms may impair cognitive capacity or be interpreted as a sign of loss of mental functioning thereby strengthening metacognitions of lack of control. A pattern of temporal relationships not consistent with the model would occur if negative emotional symptoms only gave rise to later dysfunctional metacognitions, a result that would diminish the causal status of metacognition and present a challenge to the model.

The S-REF model proposes that psychological distress (e.g., anxiety or depression symptoms) is associated with the activation of a style of thinking called the cognitive attentional syndrome (CAS). The CAS is a state of perseverative negative thinking comprised of worry, rumination, focusing on threat, and other maladaptive coping strategies that inadvertently intensify and prolong emotion responses. The CAS is hypothesized to result from metacognitions which exist in the form of knowledge, experiences, and strategies. Such components direct attention, determine thinking style and coping in response to stress cognitions and challenges (Wells, 2009). Metacognitive knowledge is relatively stable and refers to the beliefs that individuals hold about their thinking and can be categorized into positive and negative content. Positive metacognitive beliefs concern the usefulness of cognitive activities that constitute the CAS, e.g., “If I worry, I will be prepared,” while negative metacognitive beliefs concern the uncontrollability, dangerousness and importance of thoughts, e.g., “I cannot control my thinking.” Such metacognitions, especially negative beliefs are thought to impact on emotion regulation by biasing control efforts leading to perseveration of negative thinking with the effect of increasing or extending negative emotions.

A large number of studies have now demonstrated that the metacognitions predicted by the model are associated with stress symptoms, anxiety or depression (e.g., Wells and Papageorgiou, 1995; Roussis and Wells, 2008; Bennett and Wells, 2010; Yılmaz et al., 2011; Hjemdal et al., 2013; O’Carroll and Fisher, 2013; Halvorsen et al., 2015; Bailey and Wells, 2016; Fergus and Bardeen, 2016; Capobianco et al., 2018a, b). For example, Takarangi et al. (2017) conducted a longitudinal study evaluating whether metacognitive beliefs and metamemory beliefs were associated with the development and maintenance of post-traumatic stress disorder. They found that metacognitive beliefs predicted severity of PTSD symptoms after exposure to a trauma, and the maintenance of PTSD symptoms over time (time 1 to time 2).

Results from experimental manipulations of metacognitive beliefs support a causal role in negative emotion symptoms. Myers and Wells (2013) experimentally manipulated thought-event fusion beliefs (a specific type of metacognitive belief) using a fake-EEG paradigm in individuals with high and low obsessions. They found that inducing such beliefs led to OCD-like symptomology, with this effect being strongest in those with pre-existing high levels of obsessions. Capobianco et al. (2018b) also conducted an experimental manipulation of metacognitive beliefs using a similar fake-EEG paradigm. They evaluated if manipulating the belief of thought importance impacted on physiological and subjective responses to induced stress. Individuals in the experimental condition showed higher levels of negative affect and lower levels of positive affect in response to stress and maintained low positive affect at recovery. In addition to metacognitive beliefs, metacognitive strategies have also been shown to prospectively predict traumatic stress symptoms (Holeva et al., 2001; Roussis and Wells, 2008), anxiety or depression (Yılmaz et al., 2011).

Following from the S-REF model and the results demonstrating an effect of metacognitions we tested the hypothesis that metacognitive beliefs would positively predict later psychological distress measured as anxiety or depression symptoms. We did so using Structural equation modeling (SEM) as this framework allows for the use of the time ordered nature of panel data to address questions of causal orderings (Berrington et al., 2006).

## Materials and Methods

### Participants

For purposes of this study data from two samples were combined, in order to provide a sample size sufficient for SEM. Sample size and power calculations for SEM can be challenging (Wolf et al., 2013). Guidelines for SEM sample size varies; it has been suggested that a minimum sample size of 100–200 participants is required (Boomsma, 1982, 1985), but other suggestions include 5 or 10 participants per estimated parameter (Bentler and Chou, 1987), or 10 participants per variable (Nunnally, 1967). Therefore, based on the above recommendations as well as a recent evaluations using Monte Carlo simulations of sample size estimates based on model fit, suggesting a sample size of 250–300 participants (Westland, 2010; Wolf et al., 2013), we opted to combine samples from two sources to provide a sufficient sample size to conduct SEM. Two-hundred and sixty-six participants completed a questionnaire battery. In sample 1, participants (*n* = 150) were recruited from the University of Manchester. In sample 2, participants (*n* = 115) were recruited from both the University of Manchester and an online crowdsourcing website. Both samples used the same inclusion criteria; participants had to be at least 18 years of age and proficient in English. Participants ages ranged from 18 to 74 (*M* = 25.99, SD = 10.64). The sample was primarily female (213 women, 52 men). All participants from both studies completed the study using an online questionnaire software (SelectSurvey.Net). Both studies that provided data were approved by the University of Manchester Research Ethics Committee, reference 15286 (study 1) and reference 2017-2286-3683 (study 2).

### Measures

*Hospital Anxiety and Depression Scale* (HADS; Zigmond and Snaith, 1983). The HADS is a 14-item measure with two subscales; anxiety and depression. A total score can also be calculated by summating all items. Items are rated using a 4 point likert scale, where higher scores indicate greater anxiety and depression. Subscales demonstrate good internal consistency, with alpha reliabilities of 0.80 for anxiety and 0.81 for depression (Bjelland et al., 2002) and 0.86 for the total scale (Crawford et al., 2001). The scale demonstrates good reliability and validity (Herrmann, 1997; Bjelland et al., 2002).

*Meta-cognitions Questionnaire 30* (MCQ-30; Wells and Cartwright-Hatton, 2004). The MCQ-30 assesses metacognitive beliefs implicated by the S-REF model as linked to psychological vulnerability. The scale has five subscales: positive metacognitive beliefs about worry (e.g., “*Worrying helps me to solve problems*”), negative metacognitive beliefs about uncontrollability and danger (e.g., *“When I start worrying, I cannot stop”*), cognitive confidence (e.g., *“I have a poor memory”*), cognitive self-consciousness (e.g., “*I pay close attention to the way my mind works*”), and need for control (e.g., “*It is bad to think certain thoughts*”). Responses are scored on a scale ranging from 1 (do not agree) to 4 (agree very much). The scale demonstrates good convergent validity, internal consistency, and acceptable test–retest reliability (Wells and Cartwright-Hatton, 2004; Spada et al., 2008; Yılmaz et al., 2008).

### Procedure

After expressing an interest in the study participants received a link to a web site (SelectSurvey.Net) containing the participant information sheet and consent form. Following consent they were able to access the questionnaires. The questionnaire battery was distributed three times within a 2 month period. A 2 month interval was selected as this has clinical relevance; within 1 month stress symptoms normally begin to decrease, however, if they persist longer it could be indicative of a chronic or delayed stress response (deRoon-Cassini et al., 2010), therefore this interval allowed us to investigate the short and long term effects of stress within a meaningful clinical time-frame. Questionnaires were administered at day 0, day 30, and day 60.

### Statistical Analysis Plan

Statistical analyses were conducted in two steps: (1) first we examined invariance of factors over time to ensure we could include the MCQ factors in cross-lagged panel analysis, (2) we then estimated a cross-lagged panel model.

Structural equation modeling was conducted using AMOS for SPSS v 0.23 (Arbuckle, 2014) which uses the maximum likelihood (ML) method to evaluate model fit to the corresponding observed variance-covariance matrices. Model fit was evaluated using a range of fit indices including: the comparative fit index (CFI), Root Mean Square Error of Approximation (RMSEA), Standardized Root Mean Square Residual (SRMR), and Tucker Lewis Index (TLI). The following thresholds were used to assess a good model fit: CFI ≥ 0.90, RMSEA ≤ 0.06, and TLI ≥ 0.95 (Hu and Bentler, 1995), and SRMR ≤ 0.10 (Kline, 2005).

### Latent Variable Identification

In SEM, the relationships between latent variables and between latent and observed measures can be evaluated (Bollen and Noble, 2011). As latent variables cannot be directly observed, they are modeled by specifying the observed, directly measurable variables that express the underlying construct. Latent and observed variables were specified *a priori*. Metacognitive beliefs were constructed as a latent variable to allow us to evaluate the contribution of individual subscales over time while anxiety and depression were modeled as observed variables in a single model that offered the potential of controlling overlaps between anxiety and depression symptoms at each time point and any temporal relationships between these symptoms.

#### Hospital Anxiety and Depression

The HADS was modeled using the corresponding HADS subscales (anxiety and depression), as suggested by the original psychometric analysis of the scale (Zigmond and Snaith, 1983). Each subscale was treated as an observed variable rather than a latent variable as we were interested in separate overall measures of anxiety and depression rather than the contribution of the individual items to a latent general factor. The Cronbach’s alpha of the anxiety subscale was 0.81 and for the depression subscale it was 0.75.

#### Metacognitive Beliefs

The metacognition latent variable was modeled using the items corresponding to the five subscales of the MCQ-30, which is consistent with Wells and Cartwright-Hatton (2004). The scales demonstrated good internal consistency. Cronbach’s alpha for the subscales for the current study were as follows: positive metacognitive beliefs = 0.87, negative metacognitive beliefs regarding uncontrollability and danger = 0.88, cognitive confidence = 0.89, need for control = 0.80, cognitive self-consciousness = 0.85.

### Cross-Lagged Model Hypotheses

**FIGURE 1:**
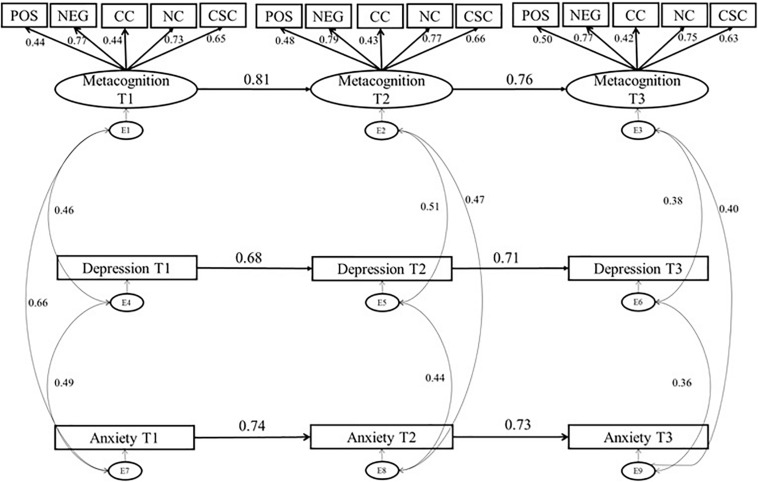
Autoregressive Model of Metacognitive Beliefs, Anxiety and Depression: standardized estimates. Solid line, significant path; dotted line, non-significant path; Pos, Positive Metacognitive Beliefs, Neg, Negative Metacognitive Beliefs Regarding Uncontrollability and Danger; CC, Cognitive Confidence; NC, Need for Control; CSC, Cognitive Self Consciousness.

We ran 3-wave structural equation models using ML estimation within AMOS V23 to investigate the longitudinal relationships between metacognitive beliefs and emotional states i.e., anxiety and depression. We began with a basic auto-regressive model, in which the latent variables have a directional effect only on themselves (Figure 1). The autoregressive model is the simplest model and acts as a reference against which to compare more complex models. We used a standard formulation of this model, in which MCQ-30 (latent variable) and anxiety and depression (observed variables) are inter-correlated within time points. In addition, the errors on individual variables that model the latents (e.g., MCQ-30 subscales) are assumed to be correlated across time-points. Inclusion of correlated errors followed the suggestion by Fornell (1983), in that inclusion is theoretically driven. We then tested the robustness of this model to violations of the assumptions before proceeding further.

Having established an appropriate autoregressive model, we next tested our hypotheses about the relationship of metacognitive beliefs to anxiety and depression through cross lagged panel analyses. Cross-lagged panel models control for contemporaneous and autocorrelations while identifying time-lagged reciprocal effects of constructs assessed repeatedly. We also accounted for the cross-lagged paths between anxiety and depression, which allowed us to evaluate and control any prospective relationships between anxiety and depression in testing if metacognition can prospectively predict anxiety or depression.

## Results

### Data Descriptives

Two-hundred and sixty-Five participants completed the study at all three time points. As less than 10% of the data was missing, mean values were imputed for missing data. Means and standard deviations of the questionnaires across time points are reported in Table 1.

**TABLE 1:** Descriptive Statistics for each measure at each test interval.

	**Time 1 (*n* = 265)**	**95% CI**	**Time 2 (*N* = 265)**	**95% CI**	**Time3 (*n* = 265)**	**95% CI**
HADS Anxiety, M (SD)	7.19 (4.05)	[6.70,7.68]	6.78 (3.89)	[6.31,7.25]	6.64 (3.92)	[6.17,7.12]
HADS Depression, M (SD)	3.24 (2.79)	[2.90,3.57]	3.29 (3.06)	[2.92,3.67]	3.37 (3.07)	[3.00,3.74]
**MCQ-30:**						
PMC About Worry, M (SD)	10.51 (3.97)	[10.03,10.99]	11.00 (3.87)	[10.52,11.46]	10.86 (3.82)	[10.40,11.32]
NMC About Uncontrollability and Danger, M (SD)	11.93 (4.72)	[11.36,12.50]	11.27 (4.48)	[10.73,11.81]	10.92 (4.30)	[10.40,11.44]
Cognitive Confidence, M (SD)	10.31 (4.15)	[9.80,10.81]	9.94 (3.89)	[9.47,10.41]	9.71 (3.83)	[9.24,10.17]
Need for Control, M (SD)	10.59 (3.82)	[10.13,11.06]	10.07 (3.42)	[9.66,10.49]	9.69 (3.59)	[9.25,10.12]
Cognitive Self Consciousness, M (SD)	14.58 (4.12)	[14.08,15.08]	13.80 (4.28)	[13.28,14.31]	13.12 (4.39)	[12.59,13.66]

*M, Mean; SD, Standard Deviation; HADS, Hospital Anxiety and Depression Scale; MCQ-30, Meta-cognitions Questionnaire 30; PMC, Positive Metacognitive Beliefs; NMC, Negative Metacognitive Beliefs.*

Pearson’s correlations (Table 2) were computed to evaluate the pattern of relationships between measures. All metacognitive beliefs were moderately to strongly positively correlated with anxiety and depression symptoms over time.

**TABLE 2:** Pearson Correlations Between Variables.

	**1**	**2**	**3**	**4**	**5**	**6**	**7**	**8**	**9**	**10**	**11**	**12**	**13**	**14**	**15**	**16**	**17**	**18**	**19**	**20**
1. Anxiety T1	–																			
2. Anxiety T2	**0.75**	–																		
3. Anxiety T3	**0.64**	**0.76**	–																	
4. Depression T1	**0.49**	**0.42**	**0.40**	–																
5. Depression T2	**0.39**	**0.53**	**0.47**	**0.70**	–															
6. Depression T3	**0.39**	**0.48**	**0.56**	**0.63**	**0.74**	–														
7. PMC T1	**0.37**	**0.34**	**0.29**	0.15^∗^	0.13^∗^	0.11	–													
8. NEG T1	**0.62**	**0.54**	**0.48**	**0.40**	**0.27**	**0.25**	**0.34**	–												
9. CC T1	**0.29**	**0.22**	**0.17**	**0.32**	**0.24**	**0.16**	**0.28**	**0.29**	–											
10. NC T1	**0.38**	**0.37**	**0.28**	**0.34**	**0.31**	**0.24**	**0.37**	**0.58**	**0.24**	–										
11. CSC T1	0.35^∗^	**0.35**	**0.28**	**0.20**	**0.20**	**0.18**	**0.32**	**0.47**	**0.16**	**0.57**	–									
12. PMC T2	**0.33**	**0.35**	**0.31**	**0.19**	**0.17**	0.15^∗^	**0.77**	**0.29**	**0.27**	**0.40**	**0.35**	–								
13. NEG T2	**0.58**	**0.66**	**0.55**	**0.33**	**0.40**	**0.33**	**0.30**	**0.77**	**0.24**	**0.53**	**0.44**	**0.35**	–							
14. CC T2	**0.26**	**0.27**	**0.22**	**0.26**	**0.34**	**0.25**	**0.18**	**0.20**	**0.71**	0.15^∗^	0.09	**0.24**	**0.32**	–						
15. NC T2	**0.34**	**0.42**	**0.30**	**0.32**	**0.43**	**0.31**	**0.31**	**0.46**	**0.27**	**0.73**	**0.45**	**0.48**	**0.60**	**0.33**	–					
16. CSC T2	**0.27**	**0.39**	**0.30**	0.14^∗^	**0.28**	**0.27**	**0.20**	**0.38**	0.14^∗^	**0.44**	**0.68**	**0.33**	**0.51**	**0.22**	**0.57**	–				
17. PMC T3	**0.32**	**0.37**	**0.37**	**0.16**	**0.17**	**0.21**	**0.71**	**0.28**	**0.26**	**0.39**	**0.29**	**0.79**	**0.31**	**0.21**	**0.38**	**0.26**	–			
18. NEG T3	**0.53**	**0.59**	**0.68**	**0.33**	**0.37**	**0.46**	**0.21**	**0.70**	**0.22**	**0.48**	**0.37**	**0.21**	**0.74**	**0.25**	**0.50**	**0.41**	**0.31**	–		
19. CC T3	**0.21**	**0.22**	**0.21**	**0.26**	**0.32**	**0.29**	**0.18**	**0.16**	**0.72**	**0.16**	0.13^∗^	**0.20**	**0.22**	**0.78**	**0.24**	**0.19**	**0.26**	**0.28**	–	
20. NC T3	**0.32**	**0.39**	**0.38**	**0.28**	**0.37**	**0.38**	**0.29**	**0.44**	**0.23**	**0.67**	**0.28**	**0.33**	**0.46**	**0.2**	**0.71**	**0.39**	**0.48**	**0.59**	**0.34**	–
21. CSC T3	**0.24**	**0.33**	**0.32**	**0.18**	**0.27**	**0.31**	0.09	**0.30**	**0.16**	**0.40**	**0.63**	0.14^∗^	**0.34**	**0.17**	**0.45**	**0.74**	**0.25**	**0.48**	**0.27**	**0.54**

*HADS = Hospital Anxiety and Depression Scale; MCQ-30 = Meta-cognitions Questionnaire 30; PMC = Positive Metacognitive Beliefs; NMC = Negative Metacognitive Beliefs; NEG = Negative Metacognitive Beliefs regarding the Uncontrollability and Danger of Worry; CC = Cognitive Consciousness; NC = Need for Control; CSC = Cognitive Self- Consciousness; T1 = Time 1; T2 = Time 2; T3 = Time 3; ^∗^ = *p* < 0.05, bold = *p* < 0.01.*

### Measurement Invariance

Measurement invariance was evaluated using the four invariance steps (configural, metric, scalar, and residual) as described by Putnick and Bornstein (2016), which coincides with those previously outlined by Widaman and Reise (1997), and Vandenberg and Lance (2000). The structure of the metacognition variable was evaluated across three time points, where we compared (a) an unconstrained model where all factor loadings and intercepts were allowed to vary freely, (b) a metric invariance model, where factor loadings were constrained equal, (c) a structural invariance model where the factor variances and covariance’s were also constrained equal, and (d) a residual invariance model where the residuals of the observed variables were also constrained equal. Measurement invariance was met for the first three steps but not for the final residual invariance model. As the item residuals are not used in the interpretation of mean differences between the latent variables, this step was not strictly necessary to show measurement invariance in this case, but was included for completeness (Vandenberg and Lance, 2000). For this reason further investigations into which residual (s) differed between the two groups were not conducted. The results of the measurement invariance analysis support the interpretation of the subsequent cross-lagged analysis as probably not unduly influenced by instability in measurement models.

### Model Testing

To evaluate if metacognitive beliefs might be a causal mechanism of anxiety and depression over time, cross-lagged panel models were used.

The initial autoregressive model (Figure 1; χ^2^ = 397.09, df = 165, *p* < 0.001) demonstrated adequate fit to the data, as the CFI and SRMR values were within the cut-offs for good fit, however, the RMSEA and TLI values were slightly above the cut-offs for good fit; CFI of 0.95, RMSEA of 0.07, SRMR of 0.08, and TLI of 0.93.

**FIGURE 2:**
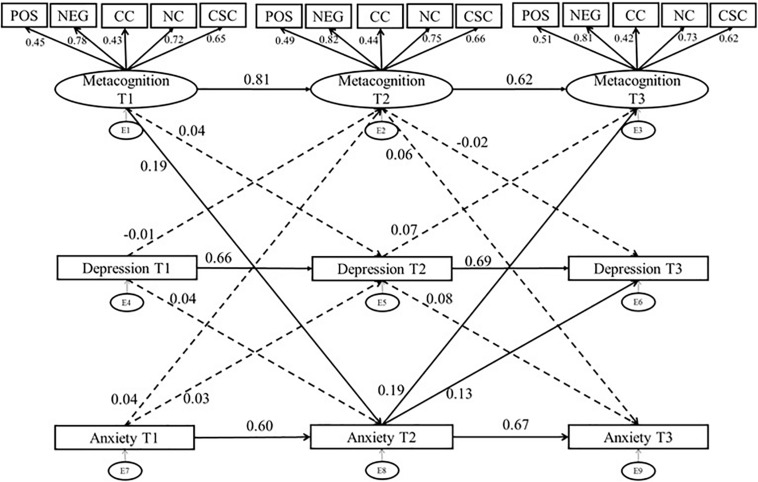
Cross Lagged Panel Model of Metacognitive Beliefs, Anxiety and Depression: standardized estimates. Solid line, significant path; dotted line, non-significant path; Pos, Positive Metacognitive Beliefs, Neg, Negative Metacognitive Beliefs Regarding Uncontrollability and Danger; CC, Cognitive Confidence; NC, Need for Control; CSC, Cognitive Self Consciousness; intercorrelations between anxiety, depression, and metacognitive beliefs were not included in the figure to increase legibility of the figure, however, are included in the analysis.

We then evaluated the full cross-lagged model (Figure 2; χ^2^ = 362.36 df = 153, *p* < 0.001), with cross-lagged paths from metacognition T1 to HADS anxiety T2 and HADS depression T2, from metacognition T2 to HADS anxiety T3 and HADS depression T3, from HADS anxiety T1 to metacognition T2, from HADS anxiety T2 to metacognition T3, from HADS depression T1 to metacognition T2, and from HADS depression T2 to metacognition T3. We also accounted for the causal associations between anxiety and depression across time, as such cross-lagged paths between HADS depression and anxiety across time-points were included. Correlations within time points between anxiety, depression, and metacognitive beliefs were also accounted for. These cross-sectional associations are not depicted in Figure 2 in order to increase legibility of the figure, however, they were included in the analysis. The cross-lagged paths significantly improved the goodness of fit (Δχ^2^ = 34.73, df = 12, *p* < 0.001; RMSEA = 0.07; CFI = 0.95, SRMR = 0.06; TLI = 0.94).

Metacognition was a positive and significant predictor of subsequent metacognitive beliefs (T1-T2: β = 0.81, *p* < 0.001, T2-T3: β = 0.76, *p* < 0.001). Similarly, anxiety was a positive and significant predictor of subsequent anxiety (T1-T2:β = 0.74, *p* < 0.001, T2-T3:,β = 0.73, *p* < 0.001) and depression predicted later depression (T1-T2:β = 0.68, *p* < 0.001, T2-T3:β = 0.71, *p* < 0.001. These results highlight the stability in metacognitive beliefs, anxiety, and depression over the testing intervals.

To evaluate if metacognitive beliefs predicted subsequent anxiety and depression and if the converse relationships applied, we examined cross-lagged regression parameters as follows:

The path from Anxiety T1 to Metacognition T2 was not significant,β = 0.04, *p* = 0.53, however, the path from Anxiety T2 to Metacognition T3 was significant, with a small beta:β = 0.19, *p* = 0.006. The finding that anxiety at T2 is predictive of subsequent metacognition is not surprising and is consistent with the metacognitive model as anxious thoughts and emotion can theoretically give rise to metacognition as previously described. However, of greater importance for the theoretical model is the path from metacognition to anxiety, here we found that metacognition at time 1 was predictive of subsequent anxiety with a small beta:β = 0.19, *p* = 0.008. Although, the path from metacognition at T2 to anxiety at time 3 was not significant,β = 0.0.06, *p* = 0.34. The beta coefficients for the paths from metacognition at T1 to anxiety at T2, and from anxiety T2 to metacognition are similar in magnitude, which raises the possibility of reciprocal causation. The result is consistent with the hypothesis that metacognitions can precede and predict negative emotion expressed as anxiety symptoms.

Depression T1 did not predict metacognition at T2 (β = −0.01, *p* = 0.82), nor did depression T2 predict metacognition T3 (β = 0.07, *p* = 0.23). Similarly, metacognition did not predict subsequent depression, metacognition T1 to depression T2 (β = 0.04, *p* = 0.61), and metacognition T2 to depression T3 (β = −0.02, *p* = 0.71). This result is not consistent with the metacognitive model applied to depression symptoms in the current sample. But it is unsurprising given that the sample had low levels of depression symptoms over time with little variation.

We also evaluated the temporal relations between anxiety and depression given that anxiety and depression commonly co-occur and may cause each other. Depression T1 did not predict anxiety at T2 (β = 0.04, *p* = 0.43), nor did depression T2 predict anxiety at T3 (β = −0.08, *p* = 0.11). While anxiety T1 did not predict depression at T2 (β = 0.03, *p* = 0.61), anxiety T2 did predict depression at T3 (β = 0.13, *p* = 0.03).

## Discussion

The current study evaluated if metacognitive beliefs prospectively predicted psychological distress symptoms measured as anxiety and/or depression. We found evidence of temporal precedence and reciprocal causation over different time lags in anxiety and the data suggested that metacognitions might be the more reliable predictor of anxiety symptoms than the converse. However, the observed effect of anxiety on metacognitions, suggests some reciprocity in these temporal relationships which is consistent with S-REF theory. However, these relationships are only indicative, we cannot rule out the possible influence of other variables that may be acting on both metacognition and symptoms. A more robust test of causal relations would require direct manipulations of metacognition and emotion in evaluating their respective causal effects.

The results for depression were different and did not appear to support any causal relationship between metacognition and mood symptoms. The results are inconsistent with other studies that show prospective relationships (Fergus and Bardeen, 2016; Ryum et al., 2017; Takarangi et al., 2017) and the prospective bivariate associations found in the current study. However, such studies and our bivariate analyses have not controlled for autoregressive and contemporaneous effects and the relationships observed may have been inflated by these factors. It is likely that the failure to find a cross-lagged relationship in the current study was impacted by the low-level of depression and lack of variability in these symptoms across time in the sample. Alternatively, it may be that the MCQ is less specific for assessing metacognitions associated with depression than with anxiety. None the less, previous studies have found that metacognitive beliefs are positively correlated with symptoms of depression and rumination both cross-sectionally (Papageorgiou and Wells, 2003; Halvorsen et al., 2015; Yılmaz et al., 2015; Huntley and Fisher, 2016) and longitudinally (Yılmaz et al., 2011).

The results add to a corpus of research supporting the idea that specific metacognitions may have a causal effect on emotion symptoms. For example, Capobianco et al. (2018b) demonstrated that induction of a metacognitive belief concerning the importance of thoughts impacted reactions to and recovery from stress exposure. For anxiety at least, the bivariate correlations in the present data set suggest that metacognitive beliefs concerning uncontrollability and danger have the strongest correlations with symptoms cross-sectionally and longitudinally, followed by metacognitions concerning “need for control” and cognitive self-consciousness. The relative strength of relationships is supported by the loadings of subscales on the latent metacognition factor where uncontrollability and need for control are the strongest contributors in the model. These findings are consistent with theory and meta-analyses, mainly of cross-sectional data, demonstrating a contribution from these metacognition domains in particular.

The limitations of the current study should, however, be considered when interpreting the findings. First, the study was primarily conducted in undergraduate students which limits the generalizability of findings, and the preponderance of women in the sample does not facilitate any examination or control of sex differences. The study did not evaluate the impact of environmental or additional factors that may influence the relationship between metacognition and symptoms and so it remains a preliminary and rudimentary test. The timescale of relationships between metacognition and emotion must also be considered. We included a time period that appeared to have clinical relevance based on the trajectory of stress responses linked more to anxiety (i.e., acute stress and PTSD) but the timescale may not be appropriate for other emotional responses to develop and remit (e.g., depression symptoms). The HADS scores were mainly below clinical cut-offs, limiting any testing of effects that might be more relevant to clinical populations. As such, further research is required to evaluate the replicability of models and pattern of results within clinical samples.

Research evaluating the temporal relationships of metacognitions and symptoms of distress are required in both clinical and non-clinical populations to determine the dynamic temporal relationships between these variables. It is recommended that such studies examine a range of time-frames with a greater number of measurement panels. They should also consider the possibility that some metacognitions may precede emotion effects whilst others may maintain symptoms leading to an increase in recovery time, especially following stress-exposure.

In conclusion, despite the limitations of the current study, the results are consistent with a pattern of temporal relationships between metacognition and anxiety that is consistent with the S-REF model. The results for depression symptoms are inconclusive and most probably affected by floor effects in the data, but an implication of the anxiety result is that anxiety might be prevented by interventions that modify specific dysfunctional metacognitive beliefs.

## Data Availability Statement

The datasets generated for this study are available on request to the corresponding author.

## Ethics Statement

The studies involving human participants were reviewed and approved by University of Manchester Research Ethics Committee, reference 15286 (study 1) and reference 2017-2286-3683 (study 2). The patients/participants provided their written informed consent to participate in this study.

## Author Contributions

All authors have contributed to the manuscript revision, read and approved the submitted version. LC was responsible for manuscript writing, data collection, entry, and analysis. CH contributed to data analysis and manuscript writing. MB was responsible for data collection and data entry. AW was responsible for study oversight and manuscript writing. AW and LC contributed to the conception and design of the study.

## Conflict of Interest

The authors declare that the research was conducted in the absence of any commercial or financial relationships that could be construed as a potential conflict of interest. The reviewer GC declared a past collaboration with one of the authors AW to the handling Editor.
